# A community study of neutralizing antibodies against SARS-CoV-2 in China

**DOI:** 10.3389/fimmu.2023.1282612

**Published:** 2023-12-07

**Authors:** Yitong Lv, Lei Huang, Junhu Wang, Hui He, Libo Song, Jia He, Lida Xu, Changyuan Yu, Ying Mei, Qi Gao

**Affiliations:** ^1^ College of Life Science and Technology, Beijing University of Chemical Technology, Beijing, China; ^2^ Health Management Center, AnQing Municipal Hospital, Anqing, Anhui, China; ^3^ Health Management Department, Shenzhen People’s Hospital, Shenzhen, China; ^4^ Health Examination Center, Central Hospital of Jin Zhou, Jinzhou, Liaoning, China; ^5^ Health Service Center, Shulan (Hang Zhou) Hospital, Hangzhou, Zhejiang, China; ^6^ Beijing Hotgen Biotech Co., Ltd, Beijing, China; ^7^ Health Management (Medical Examination) Center, The Second Affiliated Hospital of Chongqing Medical University, Chongqing, China

**Keywords:** SARS-CoV-2, neutralizing antibodies, large-scale survey, immune, China

## Abstract

**Background:**

The immune background of the overall population before and after the outbreak of SARS-CoV-2 in China remains unexplored. And the level of neutralizing antibodies is a reliable indicator of individual immunity.

**Objectives:**

This study aimed to assess the immune levels of different population groups during a viral outbreak and identify the factors influencing these levels.

**Methods:**

We measured the levels of neutralizing antibodies in 12,137 participants using the COVID19 Neutralizing Antibody Detection kit. The dynamics of neutralizing antibodies were analyzed using a generalized additive model, while a generalized linear model and multi-factor analysis of variance were employed to investigate the influencing factors. Additionally, statistical methods were used to compare neutralizing antibody levels among subgroups of the real-world population.

**Results:**

Participants who received booster doses exhibited significantly higher levels of neutralizing antibodies compared to those who received only one or two doses (p<0.001). Both elderly [22.55 (5.12, 62.03) IU/mL, 55%] and minors [21.41 (8.15, 45.06) IU/mL, 56%] showed lower positivity rates and neutralizing antibody levels compared to young adults [29.30 (9.82, 188.08) IU/mL, 62%] (p<0.001). Furthermore, the HIV-positive group demonstrated a slightly lower seropositivity rate compared to the healthy group across the three vaccination time points. Notably, three months after the large-scale infection, both the neutralizing antibody level and positivity rate in real-world populations were higher than the previous record [300 (300, 300) IU/mL, 89%; 27.10 (8.77, 139.28) IU/mL, 60%], and this difference was statistically significant.

**Conclusions:**

Increasing vaccine dosage enhances neutralizing antibody levels, resulting in greater and longer-lasting immunity. Monitoring immune levels in older individuals and those with AIDS is crucial. Additionally, the neutralizing antibodies generated from vaccination have not yet reached the threshold for achieving herd immunity, while individuals exhibit higher immune levels following a large-scale infection. These findings provide valuable insights for guiding new strategies in vaccine administration.

## 1 Introduction

The emergence of the highly contagious Corona Virus Disease 2019 (COVID-19) has posed an unprecedented global threat (1). Various methods, including vaccines, monoclonal antibodies, and convalescent plasma, have been employed to combat the SARS-CoV-2 virus by stimulating the production of neutralizing antibodies (NAbs) (2, 3). NAbs are crucial in protecting the human body by binding to the virus surface protein therby preventing its interaction with host cell receptors (4). The production of NAbs, induced through either vaccination or natural infection, is pivotal in controlling viral infections. However, as SARS-CoV-2 adapts to its human host, it undergoes genetic evolution, resulting in the emergence of multiple variants over time. These variants may exhibit enhanced transmissibility or virulence, show reduced neutralization by antibodies acquired through natural infection or vaccination, evade detection, or decrease the effectiveness of therapeutics or vaccines (1, 5). The duration of immunity conferred by neutralizing antibodies obtained through vaccination remains uncertain, especially in individuals with immunodeficiencies. Therefore, evaluating NAb levels is valuable for determining the duration of the protective humoral response and for selecting donors for convalescent plasma therapy (6).

Assessing NAb titers is essential in determining their ability to neutralize the virus, facilitate virus clearance, and confer long-lasting protective immunity (6). Multiple studies have consistently demonstrated that following vaccination, serum antibody levels initially rise, reach a peak and subsequently decline. For example, one study found that after the second dose of an inactivated vaccine, the positivity rate of NAbs reached a peak in the second month and gradually declined over time (7). Similarly, another study observed a rapid increase in serum antibody levels within two weeks after the administration of the second and third vaccine doses, followed by a peak and subsequent decline (8). Moreover, a community-based cohort study in Germany reported that NAbs were detected in only onethird of positive immune assay results, and that their levels decayed relatively quickly (9). NAbs induced by vaccines or prior infection play a pivotal role in controlling SARS-CoV-2 (10). Longitudinal studies examining the kinetics of the neutralizing antibody response in large populations can provide critical insights into the durability of immunity (11).

Moreover, NAb levels are influenced by various factors. Some research has indicated that the neutralizing potency of antibodies diminishes with age, with similar levels between sexes (12). Conversely, another study of 3,808 participants found substantially lower neutralizing antibody titers among men compared to women, as well as in individuals aged 65 years or older compared to those aged 18 to less than 45 years, six months after receiving the second vaccine dose (13). Considering these factors, it is important to examine the distribution of NAbs across different age and gender groups, as increasing sample size can enhance the reliability and generalizability of conclusions.

In addition to studying the immune responses in immunocompetent individuals, it is also crucial to investigate the immune responses in immunocompromised populations, such as individuals with AIDS. These individuals may exhibit altered or attenuated immune responses to SARS-CoV-2 infection compared to immunocompetent individuals (14). Research findings indicate that individuals with AIDS demonstrate comparable cellular and humoral immune responses to COVID-19 vaccinations compared to healthy control groups after receiving two doses (15). Similarly, studies have also shown that individuals with AIDS, can achieve similar NAb positivity rates similar to HIVnegative populations with inactivated vaccinations, although their overall response is weaker (16). However, the heterogeneity of immune dysfunction in these individuals implies that some may display variable, weak, or diminished vaccine-induced immune responses (17). Thus, it remains crucial to evaluate the impact of vaccination on NAb levels in AIDS patients is crucial for clinical practice insights.

In this study, we conducted a large-scale multi-center analysis to investigate the immune status of the population before the nationwide infections in China between December 2022 and January 2023. We measured NAb levels from different centers and examined the variations over time after each dose. Additionally, we analyzed the differences in NAb levels between AIDS patients and healthy individuals following vaccination, as well as the influence of age and gender on the levels. Our findings provide meaningful insights into the real-world immune barrier prior to the large-scale infections. Furthermore, after the large-scale infections, we conducted a community-based survey in a small population in February 2023 to analyze the changes in neutralizing antibody levels pre- and post- infection. The results of this study offer empirical evidence to inform national immunization policies and epidemic control strategies, as well as booster shot planning.

## 2 Methods

### 2.1 Study population

All the blood samples were collected from five medical centers: AnQing Municipal Hospital, Shenzhen People’s Hospital, Central Hospital of Jin Zhou, Shulan (Hang Zhou) Hospital, and The second affiliated hospital of Chongqing Medical University.

A total of 6,100 vaccinated samples, with no history of SARS-CoV-2 infection, were collected before the national-wide infection during December 2022 to January 2023. These samples were obtained from individuals vaccinated with an inactivated vaccine, primarily sourced from Sinovac or Sinopharm. Sample collection spanned thirteen months, from March 2021 to April 2022. The samples were categorized as follows for further analysis: (1) 100 random samples for accessing the accuracy of the COVID-19 Neutralizing Antibody Detection kit; (2) 4,920 samples in a cross-sectional survey, with 441, 2,757, and 1,722 samples representing individuals who received one, two, and three vaccine doses, respectively; (3) 786 samples from 131 individuals were tested for NAb levels at six time points post-vaccination; (4) 294 samples to investigate NAb levels between the AIDS and healthy groups.

Additionally, 6,699 samples with unknown vaccination status were also collected before the national-wide infection for assessing the NAb levels in the real world. Furthermore, 100 samples were collected in February 2023 from the same centers for assessing changes in NAb levels post-large-scale infection.

This study was conducted with the approval of the Institutional Ethics Committee of Peking Union Medical College Hospital.

### 2.2 Experiment methods

A recombinant backbone plasmid, prVSV△G-S2, containing the spike protein gene sequence of SARS-CoV-2 (sequence: RVQPTESIVRFPNITNLCPFGEVFNATRFASVYAWNRKRISNCVADYSVLYNSASFSTFKCYGVSPTKLNDLCFTNVYADSFVIRGDEVRQIAPGQTGKIADYNYKLPDDFTGCVIAWNSNNLDSKVGGNYNYLYRLFRKSNLKPFERDISTEIYQAGSTPCNGVEGFNCYFPLQSYGFQPTNGVGYQPYRVVVLSFELLHAPATVCGPKKSTNLVKNKCVNFNFNGLTGTGVLTESNKKFLPFQQFGRDIADTTDAVRDPQTLEILDITPCS), was constructed. Recombinant vaccinia virus vTF7-3 was used to infect BHK21 cells, achieving a cell fusion efficiency of approximately 80%. The main plasmid prVSV△G-S2 and the helper plasmids pBS-N, pBS-P, pBS-G, and pBS-L were co-transfected into BHK21 cells at specific ratios. After 48 hours of transfection, the culture supernatant was collected, filtered, and added to BHK21 cells pre-transfected with the helper plasmid pCAGGS-G. Blind passaging was performed for 2-3 generations using this method. Subsequently, the supernatant from BHK21 cells was used to infect VeroE6 cells for blind passaging for several generations, followed by filtration of the supernatant. The collected recombinant virus was concentrated using a virus concentration kit and resuspended in an appropriate volume of PBS solution. The concentrated virus was stored at -80°C. To determine the viral titer of the recombinant virus, VeroE6 cells were seeded in a 96-well plate until they reached approximately 90% confluency. The concentrated virus suspension was diluted 10-fold with PBS, generating 12 serial dilutions. Each dilution of the virus suspension was inoculated into the VeroE6 cells at a volume of 100 μL per well, with 6 replicate wells for each dilution. The cells were cultured at 37°C for 3 days, and the cell status was observed daily. The viral titer was calculated using the Reed-Muench method (18).

A 5 mL sample of whole blood was collected from each participant and centrifuged at 4000 RPM for 10 minutes. The separated serum samples were stored below -20°C and analyzed within two months, with no more than three freeze-thaw cycles. The COVID-19 Neutralizing Antibody Detection kit (Beijing Hotgen Biotech Co., Ltd.) was used following the manufacturer’s instructions. The kit utilizes a competition method based on magnetic particle-based chemiluminescence immunoassay technology to detect NAbs against SARS-CoV-2 in blood samples. During the test, the sample was incubated with alkaline phosphatase (ALP)-labeled S-RBD antigen, leading to the formation of a complex of neutralizing antibodies and S-RBD antigen. Subsequently, biotin-labeled receptor protein ACE2 and streptavidin-coated magnetic microbeads were added. The ACE2-S-RBD antigen complex was immobilized on the magnetic microbeads through specific binding between biotin and streptavidin. The resulting complex was separated from unbound substances using a magnetic field and subjected to washing to remove any unbound materials. Afterward, a chemiluminescent substrate was added to the immune complex. The chemiluminescence signal generated by the enzyme reaction was detected using an automated chemiluminescence immunoassay analyzer (C2000). The intensity of the detected luminescence is inversely correlated with the concentration of neutralizing antibodies against SARSCoV-2 in the sample. The analyzer can calculate the S/CO (signal-to-cutoff) ratio of the novel coronavirus neutralizing antibodies in the sample, and the results are interpreted based on the established criteria. In the case of qualitative kits, a result is considered positive when S/CO < 1.00. The neutralizing antibody levels were measured using the international standard serum for SARS-CoV2 neutralizing antibodies, calibrated in IU/mL units, as designated by the NIBSC WHO code: 20/136. This international standard material, obtained from NIBSC, has a known antibody concentration of 250 IU per ampoule. We established a calibration curve using this standard and quantified the antibody concentrations in the samples. Accordingly, the recorded neutralizing antibody levels were converted to international units (IU) per milliliter (19). For the quantitation kit, a positive result is determined when the concentration of NAbs in the sample exceeds 17.6 IU/mL. To account for subsequent viral mutations, the S-RBD antigen used in the test was also appropriately substituted based on the corresponding mutant strains to ensure the accuracy of detection.

### 2.3 Statistical analysis

Statistical analyses were performed using R version 4.1.2. Generalized linear models (GLM) and multifactor analysis of variance (multi-factor ANOVA) were employed for data fitting. The Spearman correlation coefficient was utilized to assess the correlation between variables. Group comparisons were conducted using the Wilcoxon signed-rank test, Kruskal-Wallis test, Fisher’s exact test, and Pearson’s Chi-squared test. A p-value of less than 0.05 was considered statistically significant.

## 3 Results

### 3.1 Diagnostic accuracy of the COVID-19 Neutralizing Antibody Detection kit

**Figure 1:**
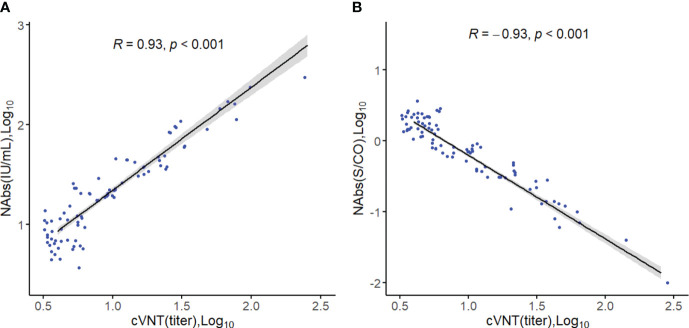
Spearman correlation analysis. **(A)** Correlation between the level of neutralizing antibodies (Log10) and viral neutralizing titers. **(B)** Correlation between neutralizing antibody levels (S/CO, Log10) and viral neutralizing titers.

The accuracy of the COVID-19 Neutralizing Antibody Detection kit was assessed in a sample of 100 vaccinated individuals. The results in **Figure 1** demonstrated a significant correlation between the levels of NAbs measured by the kit and the neutralizing titers of viruses evaluated using cVNT experiments (R=0.93, p<0.001, **Figure 1A**). Furthermore, the NAbs (S/CO) showed a significant correlation with the neutralizing titer of viruses (R=-0.93, p<0.001, **Figure 1B**), indicating the high accuracy of the kit in detecting NAbs. Additionally, there was strong consistency between the levels of NAbs and the NAbs (S/CO) (Kappa=0.960). To assess the agreement between the two measurement methods, we also conducted a Passing-Bablok regression analysis. The analysis revealed a relationship between the measurements, with a slope of 0.75 (95% confidence interval: 0.66-0.83) and an intercept of -0.03 (95% confidence interval: -0.13-0.06).

### 3.2 Study on the level of neutralizing antibodies after COVID-19 vaccination

A total of 4,920 samples with vaccination information were included in the analysis to investigate the kinetics of NAb levels after COVID-19 vaccination. This sample comprised 2,758 males (57%) and 2,162 females (43%) with a median age of 41 years (range 18-88). Detailed sample characteristics are presented in **Table 1**.

**Table 1:** Age and gender distribution of participants.

Characteristic	First dose (N = 441^1^)	Second dose (N = 2,757^1^)	Third dose (N = 1,722^1^)
Gender			
Female	168 (38%)	1,105 (40%)	889 (52%)
Male	273 (62%)	1,652 (60%)	833 (48%)
Age	50 (43, 56)	43 (34, 53)	37 (31, 44)
≥55	149 (34%)	594 (22%)	117 (7.0%)
18~55	292 (66%)	2,163 (78%)	1,605 (93%)
Time since vaccination	17 (8, 34)	78 (51, 137)	130 (109, 140)

^1^n (%); Median (IQR).

**Figure 2:**
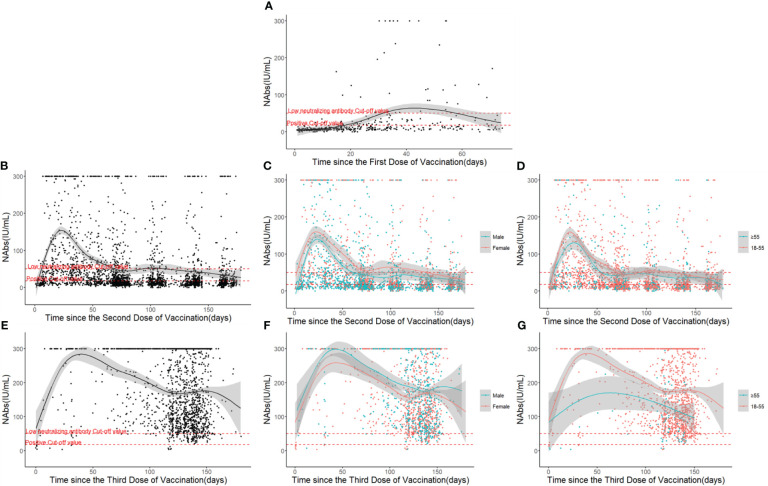
Neutralizing antibody response and duration following COVID-19 vaccinations over time: **(A)** After the first dose. **(B)** After the second dose. **(C)** Different gender groups after the second dose. **(D)** Different age groups after the second dose. **(E)** After the third dose. **(F)** Different gender groups after the third dose. **(G)** Different age groups after the third dose.

The factors influencing the levels of NAbs after vaccination were analyzed using a multi-factor ANOVA. For individuals who received one dose of the vaccine, the levels of NAbs primarily varied with time. In contrast, for those who received two or three doses of the vaccine, gender, age, and the time since vaccination all had an impact on the level of NAbs (**Table 2**). Furthermore, the neutralizing antibody kinetics after different doses of the COVID-19 vaccination were analyzed using a generalized additive regression model. Regression analyses of neutralizing antibody levels were also performed on samples that received two or three doses of the vaccine, grouping them by gender and age (**Figure 2**).

**Table 2:** Multi-factor ANOVA for neutralizing antibodies: gender, age, and time since vaccination.

Dose	Item	F	P value
First dose	Gender	1.047	0.307
	Age	0.042	0.838
	**Time since Vaccination**	2.628	**<0.001**
Second dose	**Gender**	22.558	**<0.001**
	**Age**	4.842	**0.028**
	**Time since Vaccination**	3.870	**<0.001**
Third dose	**Gender**	10.520	**0.001**
	**Age**	28.259	**<0.001**
	**Time since Vaccination**	2.269	**<0.001**

P < 0.05 was considered statistically significant.

The results revealed that the level of NAbs reached an initial peak after each shot and subsequently decreased with time. Following the first dose of vaccination, the level of NAbs rose gradually until it reached a peak at arount 40 days, generally at a relatively low level (17.6 IU/mL ≤ NAbs ≤ 50 IU/mL) (**Figure 2A**). After the second dose of vaccination, the level of NAbs increased rapidly, peaking between the 20th and 30th day, but then declined to a low level around the 70th day (**Figure 2B**). The NAb response was strongest in individuals who received three doses of the vaccine, with the response lasting for 6 months and peaking at 300 IU/mL before dropping to approximately half of the peak level (**Figure 2E**).

While the dynamic curve of neutralizing antibody levels over time was not significantly influenced by gender, there were gender-based differences in the overall level of NAbs (**Figures 2C, F**). Participants aged above 55 took longer to reach the peak level of NAbs and generally had lower levels, with these differences more pronounced in the samples that received three doses of the vaccine (**Figures 2D, G**).

### 3.3 Dynamic monitoring of neutralizing antibodies

**Figure 3:**
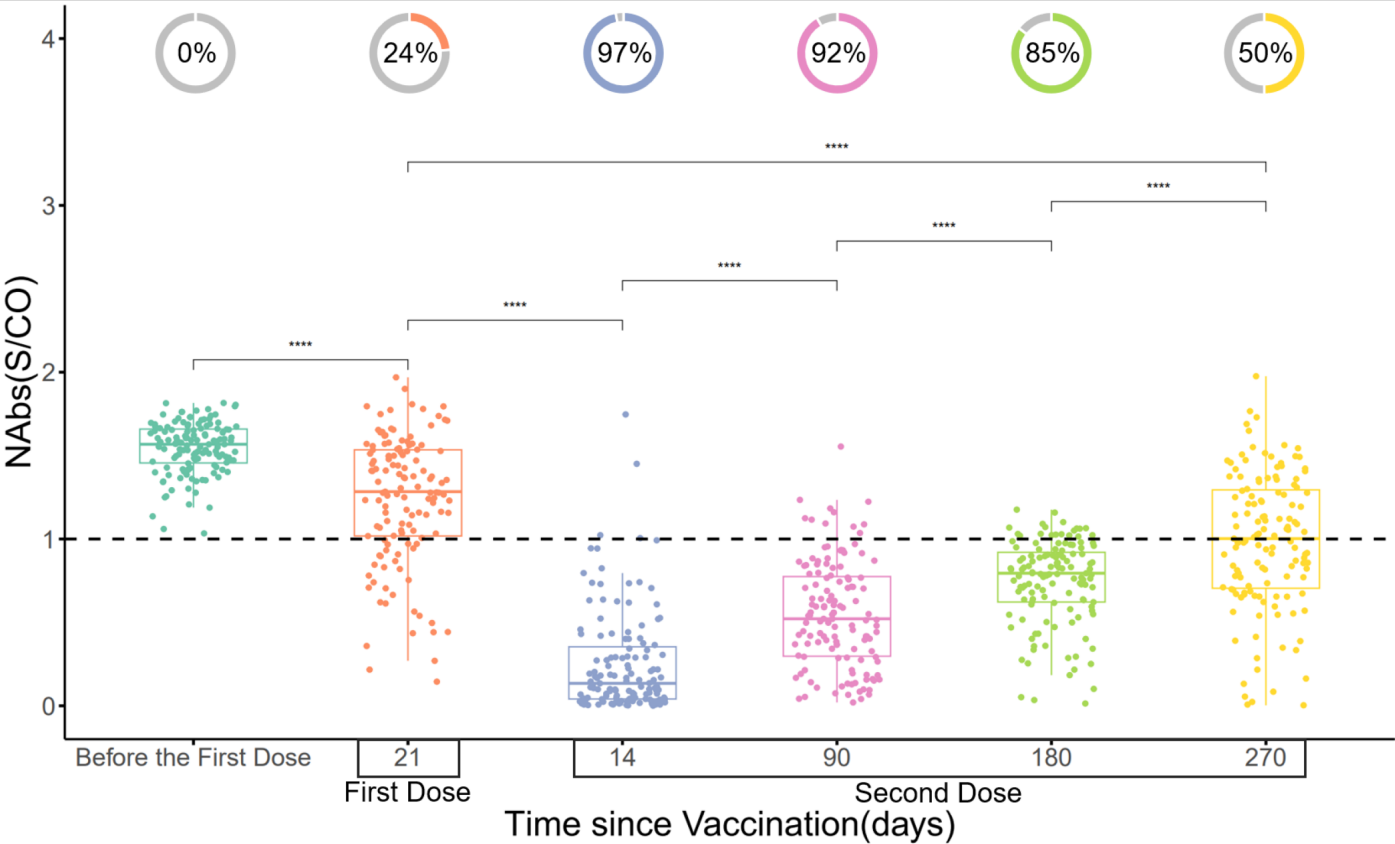
Changes in NAbs (S/CO) over time. The donut charts illustrate the proportion of individuals with positive NAbs (NAbs with S/CO values less than 1 are classified as positive). Statistical analysis was performed using the Wilcoxon signed-rank test (**** indicate p < 0.0001).

The protective efficacy of the vaccine was evaluated by dynamically monitoring the NAbs (S/CO) of 131 participants who received the COVID-19 vaccine at six time points: before the first dose of vaccination, the 21st day after the first dose, the 14th, 90th, 180th, and 270th day after the second dose. The results demonstrated that the maximum positivity rate of NAbs was achieved on the 14th day after the second dose (approximately 97%), gradually decreasing and dropping to 50% on the 270th day after the second dose, which still remained higher than the 24% positivity rate observed on the 21st day after the first dose (p<0.001) (**Figure 3**).

### 3.4 Comparison of the level of NAbs in AIDS patients and healthy individuals

**Figure 4:**
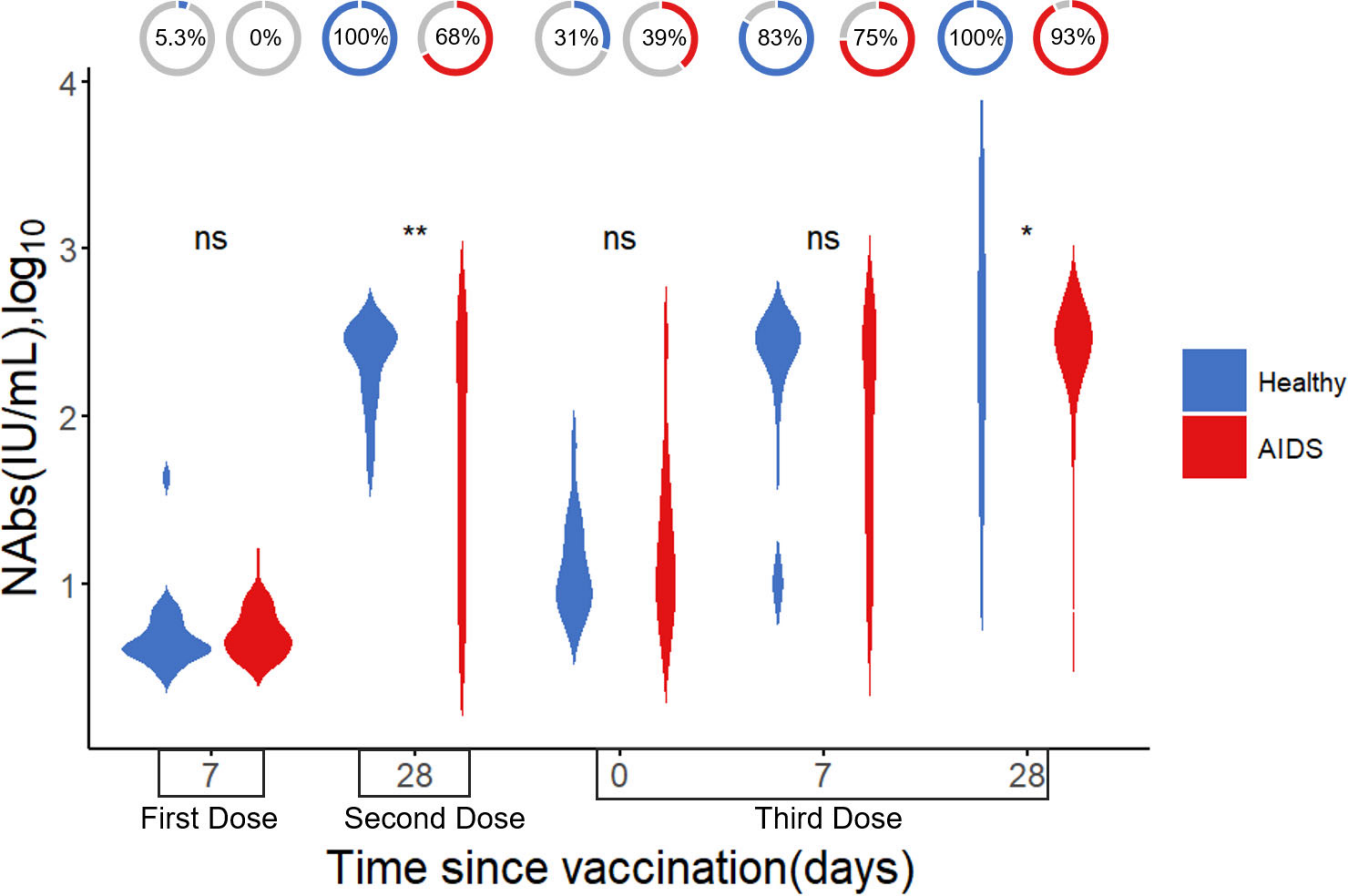
Difference in NAb levels (log_10_) after vaccination between AIDS and healthy groups. The donut charts illustrate the proportion of individuals with positive NAbs. Statistical analysis performed using the Wilcoxon signed-rank test (ns, not significant; *, ** indicates p < 0.05, and <0.01, respectively).

Neutralizing antibody levels were measured in 49 AIDS patients and 45 healthy individuals at five different time points after COVID-19 vaccination. Detailed information is presented in **Table 3**. The results indicated that neutralizing antibody levels were comparable between the healthy group and the AIDS group after the first vaccine dose. However, after the second and third doses, NAb levels were significantly lower in the AIDS group. Nonetheless, 28 days after the third dose, both groups displayed an average neutralizing antibody level of around 300 IU/mL with only minor variations in NAb positivity rates observed between the groups after vaccination. It is worth noting that the NAb response in the AIDS group exhibited heterogeneity in comparison to the response observed in healthy individuals. These outcomes are visually represented in **Figure 4** and summarized in **Table 4**.

**Table 3:** Characteristics of AIDS patients and healthy individuals.

Characteristic	AIDS patients (N = 49^1^)	Healthy individuals (N = 45^1^)
Gender		
Female	39 (80%)	34 (76%)
Male	10 (20%)	11 (24%)

^1^n (%); Median (IQR).

**Table 4:** NAb Levels (IU/mL) and positivity rates in AIDS and healthy groups.

		First dose	Second dose		Third dose	
Time since Vaccination		7	28	0	7	28
The level of NAbs*^1^*	AIDS	4.85 (3.87, 6.41)	50.72 (11.61, 300.00)	13.66 (8.00 37.78)	80.94 (21.92, 300.00)	300.00(300.00, 300.00)
	Healthy	4.19 (3.95, 5.61)	300.00 (149.71, 300.00)	10.63 (8.06, 21.25)	300.00 (117.23, 300.00)	300.00(300.00, 300.00)
p-value*^2^*		0.2	**<0.01**	0.2	0.2	**<0.05**
NAbs positive*^3^*	AIDS	0 (0%)	21 (68%)	13 (39%)	24 (75%)	25 (93%)
	Healthy	1 (5.3%)	16 (100%)	11 (31%)	25 (83%)	32 (100%)
p-value*^4^*		0.3	**<0.01**	0.5	0.5	0.2

^1^Median (IQR).

^2^Wilcoxon rank sum test.

^3^n (%).

^4^Fisher’s exact test or Pearson’s Chi-squared test.

P < 0.05 was considered statistically significant.

### 3.5 Study on the level of neutralizing antibodies before and after the large scale infection

To understand the level of NAbs in real-world populations, a total of 6,699 samples from individuals with an unknown vaccination status prior to the large-scale infection and tested for neutralizing antibody levels, which included 2,458 males (37%) and 4,241 females (63%), with a median age of 42 years (range 0-96). The overall neutralizing antibody levels in this population were 27.10 (8.77, 139.28) IU/mL, with a 60% positivity rate (**Table 5**). There was no significant difference in the neutralizing antibody levels or positivity rate of NAbs between males and females (p>0.05). However, there were lower neutralizing antibody levels and positivity rates in both the elderly and minors, with a significant difference observed (p<0.05).

**Table 5:** The level (IU/mL) and positivity rates of NAbs.

	The level of NAbs*^1^*	P value*^2^*	NAbs positive *^3^*	P value*^4^*
Gender				
Female, N=4241	26.83 (9.10, 124.82)	0.8	2,564 (60%)	>0.9
Male, N=2458	27.58 (8.18, 162.40)		1,487 (60%)	
Age				
≤18, N= 124	21.41 (8.15, 45.06)	**<0.001**	69 (56%)	**<0.001**
18~55, N= 4549	29.80 (10.10, 209.40)		2852 (63%)	
≥55, N= 2026	23.85 (5.90, 72.19)		1130 (56%)	
Overall	**27.10 (8.77, 139.28)**		**4,051 (60%)**	

^1^Median (IQR).

^2^Wilcoxon or Kruskal-Wallis rank sum test.

^3^n (%).

^4^Pearson’s Chi-squared test.

P < 0.05 was considered statistically significant.

**Figure 5:**
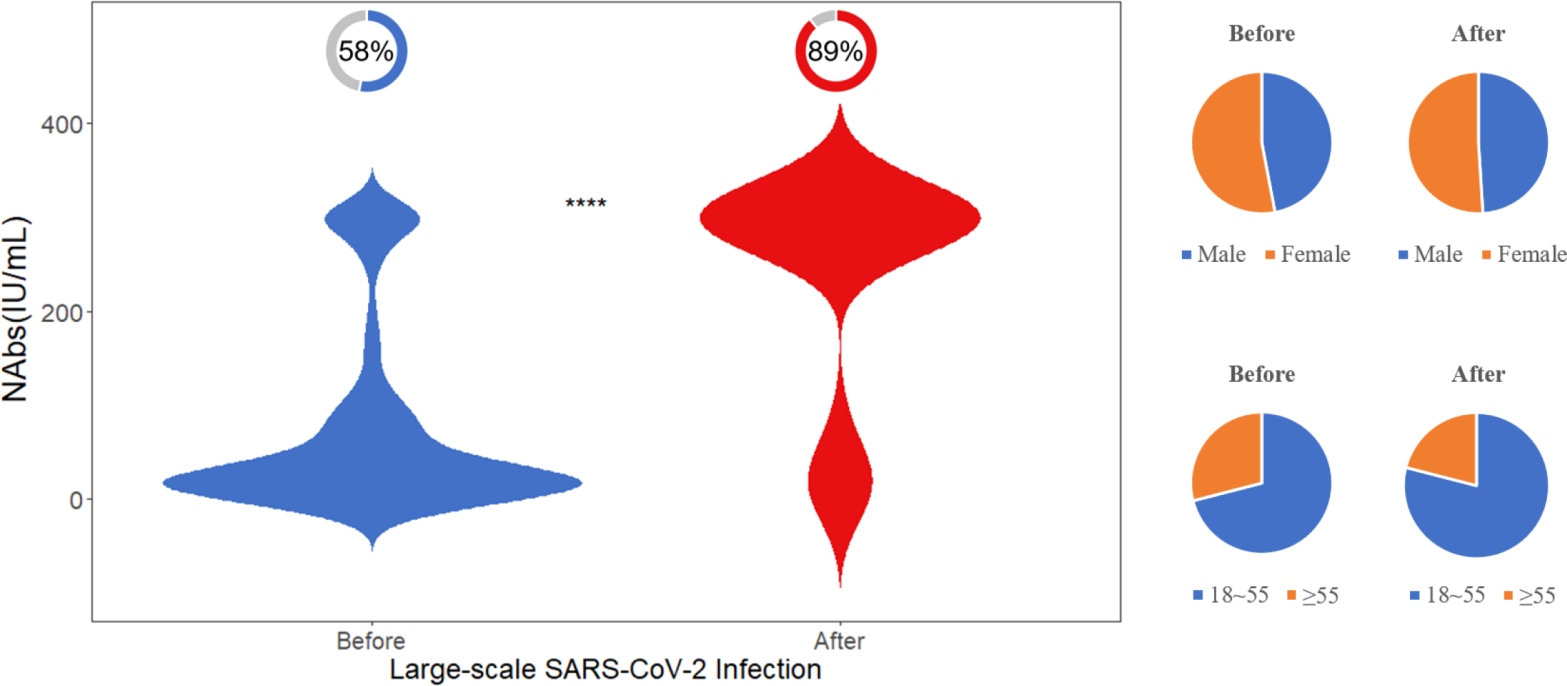
Difference in NAb levels before and after the large-scale SARS-CoV-2 infection. The donut charts illustrate the proportion of individuals with positive NAbs. Statistical analysis performed using the Wilcoxon signed-rank test (**** indicates p <0.0001).

To compare the changes in neutralizing antibodies between populations before and after a large-scale SARS-CoV-2 infection, we collected 100 samples within three months after the infection and randomly selected 100 pre-infection samples with a similar gender and age distribution for comparison. As displayed in **Figure 5**, after the large-scale infection, the positivity rate of NAbs was 89%, and the level of NAbs was 300 (300, 300) IU/mL, which was significantly higher than the pre-infection population (p<0.001).

## 4 Discussion

The COVID-19 pandemic has persisted for over three years, and the emergence of new variants has posed significant challenges to epidemic control. NAb levels are critical in assessing herd immunity and guiding public health responses (20). In this study, we aimed to investigate the real-world population-level NAb levels in China prior to the large-scale infections (from December 2022 to January 2023) caused by the Omicron SARS-CoV-2 variant and compared them with NAb levels after the infection. Additionally, we analyzed the factors influencing NAb levels, the kinetics of protection duration, and compared changes in NAb levels following vaccination between individuals with AIDS and healthy individuals to inform future vaccination strategies.

We initially examined the kinetics of NAb levels after vaccination and observed a gradual increase in NAb levels and duration with each vaccine dose, indicating the development of long-lasting immune memory. While NAb levels generally decline over time, our findings revealed that the peak NAb level after the third vaccine dose wsa higher compared to that after the second dose, which aligns with previous research (8, 21, 22).

Several factors influence NAb levels, including vaccination time, gender, age, vaccine type, and ethnicity. Age was identified as a crucial factor affecting NAb levels, with lower levels observed in the elderly compared to younger individuals. This phenomenon was more pronounced in those who received the third vaccine doses, consistent with previous studies (23). Minor differences in Nab levels were also observed between genders. Special attention should be given to enhancing protection among older adults.

Our findings suggest that even after receiving a third vaccine dose, the NAb positivity rate in the AIDS group was slightly lower compared to the healthy group, aligning with previous studies indicating a diminished response to certain vaccines among individuals with AIDS (24). Although a third vaccine dose elicited a rapid and stronger NAb response in AIDS patients, the overall immunogenicity in individuals with AIDS is lower when compared to healthy controls (25, 26). In addition, the weak response of immunocompromised individuals to the Omicron variant (27) also highlights the heterogeneity in the antibody response within individuals with compromised immune function. This heterogeneity may lead to insufficient protection against COVID-19 disease even after vaccination (17). This weaker antibody response to the virus post-vaccination can potentially increase their risk of infection. These considerations should be taken into account when developing vaccine strategies for individuals with AIDS.

Prior to the large-scale-infection, the real-world NAb positivity rate in the population of China was 60%, with average levels of 27.10 (8.77, 139.28) IU/mL. However, some individuals exhibited inadequate neutralizing antibody levels, and SARS-CoV-2 variants with immune escape capabilities emerged. Immunological imprinting generally refers to the impact of prior exposure on subsequent immune responses, ultimately providing protection against related viruses (28). However, it is important to note that immunological imprinting can also result in a narrower focus of our immune system on previously encountered epitopes, limiting its ability to effectively recognize and respond to new variant strains, leading to a potential reduction in antibody diversity (29). This phenomenon of immunological imprinting not covering other variant strains may have played a role in the subsequent large-scale infections observed (28). Moreover, the repeated administrations of inactivated vaccines may induce a strong immune response against the original strain but could potentially reduce the response to new strains, which may have contributed to the subsequent large-scale infection (30). Other literature has also indicated that the administration of heterologous vaccines has better immunogenicity (31–34). After the large scale infection, the NAb positivity rate reached 89% with levels averaging 300 (300, 300) IU/mL, indicating the establishment of an effective immune barrier and prevention of a second large-scale transmission. Nevertheless, there are limitations in comparing changes in NAb levels between populations before and after the large-scale infection. These limitations include the relatively small number of samples collected after the infection and the lack of vaccination information for the 6,699 samples used to measure NAb levels in the real-world population. Additionally, another limitation is that the antigen used in the assay kit, although continuously updated and monitored for performance against new strains, may still exhibit performance biases when measuring different strain variants. However, these limitations do not undermine the conclusions drawn from our study.

In conclusion, our study provides important insights into the levels and kinetics of NAbs in largescale real-world settings, as well as the factors influencing their levels. Our findings highlight the influence of age and AIDS status on NAb levels, emphasizing the need to increase vaccination rates among older adults and improve vaccine responses in AIDS patients. Furthermore, our study underscores the importance of achieving a high NAb positivity rate to establish an effective immune barrier and prevent large-scale virus transmission. However, in light of recent findings in COVID vaccinations and the phenomenon of immunological imprinting, it is crucial to consider alternative strategies to enhance vaccine efficacy and address the limitations of current approaches. One potential avenue is to explore the development of vaccines targeting new variant strains that differ significantly from the original strain, thus avoiding immunological imprinting and improving cross-protection against emerging variants. By incorporating these insights into new vaccination strategies, we can contribute to the fight against the COVID-19 pandemic.

## Data availability statement

The original contributions presented in the study are included in the article/supplementary material. Further inquiries can be directed to the corresponding authors.

## Ethics statement

Ethical approval was not required for the studies on animals in accordance with the local legislation and institutional requirements because only commercially available established cell lines were used. Written informed consent was obtained from the minor(s)’ legal guardian/next of kin for the publication of any potentially identifiable images or data included in this article. This study was approved by the Institutional Ethics Committee of Peking Union Medical College Hospital (JS-3449D).

## Author contributions

YL: Formal Analysis, Writing – original draft, Writing – review & editing, Validation. LH: Methodology, Validation, Writing – review & editing. JW: Data curation, Writing – review & editing. HH: Data curation, Writing – review & editing. LS: Data curation, Writing – review & editing. JH: Data curation, Writing – review & editing. LX: Investigation, Writing-review & editing. CY: Conceptualization, Supervision, Writing-review & editing. YM: Conceptualization, Supervision, Writing – review & editing. QG: Conceptualization, Supervision, Writing – review & editing.
